# Loffler endocarditis as an initial manifestation of lung adenocarcinoma: A case report

**DOI:** 10.1097/MD.0000000000046394

**Published:** 2025-12-12

**Authors:** Hongmei Yao, Yubin Chen, Chao Wu, Zijing Wang, Feng Bai

**Affiliations:** a Department of Cardiology, The First Hospital of Shanxi Medical University, Taiyuan, Shanxi Province, China; b Shanxi Medical University, Taiyuan, Shanxi Province, China

**Keywords:** hypereosinophilic syndrome, Loffler endocarditis, lung adenocarcinoma, treatment

## Abstract

**Rationale:**

Löffler endocarditis, a rare form of hypereosinophilic syndrome (HES), is characterized by transient left ventricular endomyocardial thickening, intracardiac thrombi, and eosinophilic infiltration. Its occurrence as a paraneoplastic manifestation of solid tumors, particularly lung adenocarcinoma, is exceedingly rare and underrecognized.

**Patient concerns:**

A 75-year-old male smoker presented with progressive dyspnea (New York Heart Association Class III) and bilateral leg edema.

**Diagnoses:**

He had no history of asthma, allergy, or parasitic infection. Physical examination revealed jugular venous distention and bilateral crackles. Laboratory tests showed marked hypereosinophilia (15.29 × 10^9^/L). Transthoracic echocardiography demonstrated endomyocardial thickening, reduced left ventricular compliance, and mobile intracardiac thrombi. Computed tomography angiography revealed a spiculated left upper lobe lung mass with mediastinal lymphadenopathy.

**Interventions:**

Symptoms developed over 4 weeks. Hypereosinophilia and cardiac abnormalities were identified at presentation. The lung mass was detected on initial imaging; histopathology confirmed adenocarcinoma (biopsy-proven). Despite anticoagulation and heart failure therapy, the patient declined corticosteroids and oncologic treatment. Clinical deterioration occurred within 2 weeks, leading to death. The patient received guideline-directed medical therapy for heart failure (beta-blocker, angiotensin-converting enzyme inhibitor, diuretics), anticoagulation (low-molecular-weight heparin), and diagnostic bronchoscopic biopsy. Corticosteroids and chemotherapy were recommended but refused by the family.

**Outcomes:**

The patient’s condition rapidly worsened due to progressive heart failure and untreated malignancy.

**Lessons:**

This case highlights the importance of considering occult malignancy in unexplained HES with cardiac involvement. It is the first reported case of Löffler endocarditis as a paraneoplastic manifestation of lung adenocarcinoma. Limitations include the lack of postmortem examination and the inability to initiate immunosuppressive or antitumor therapy due to patient refusal.

## 1. Introduction

Hypereosinophilic syndrome (HES) is defined by sustained eosinophilia (>1.5 × 10⁹/L) with end-organ damage, most commonly affecting the heart, lungs, and nervous system.^[1]^ Löffler endocarditis, a subtype of HES, progresses through necrotic, thrombotic, and fibrotic phases, often leading to restrictive cardiomyopathy and thromboembolic complications.^[2]^ While HES is frequently linked to hematologic malignancies or parasitic infections, its association with solid tumors, such as lung adenocarcinoma, is exceedingly rare. We reported a case of lung adenocarcinoma initially presented with Loffler Endocarditis as part of paraneoplastic syndrome. To our knowledge, this is the first reported case of Löffler endocarditis as a paraneoplastic manifestation of lung adenocarcinoma.

## 2. Case report

A 75-year-old male patient presented with bilateral lower limb edema and exertional dyspnea of unknown origin in October 2022, which he initially neglected. At that time, no formal medical evaluation was sought. By January 2023, his symptoms worsened, characterized by significantly reduced exercise tolerance, orthopnea, fatigue, and oliguria.

He first sought care at Shanxi Provincial Hospital of Traditional Chinese Medicine in early January 2023, where he received unspecified oral herbal therapy. The treatment showed no clinical improvement over the following 2 weeks, prompting referral to the First Hospital of Shanxi Medical University on January 2023. Since symptom onset, the patient reported poor mental status, appetite, and sleep quality, along with recent constipation and decreased urine output, while his weight remained stable. His medical history was unremarkable for hypertension, diabetes, or cardiovascular/cerebrovascular diseases. He had a 40-year smoking history (20 cigarettes/day).

Upon admission, initial laboratory evaluation revealed marked hematologic abnormalities: White blood cells: 56.7 × 10⁹/L, red blood cells: 3.35 × 10¹²/L, Hemoglobin: 108 g/L, Absolute monocyte count: 1.98 × 10⁹/L, Absolute neutrophil count: 37.68 × 10⁹/L, Absolute eosinophil count: 15.29 × 10⁹/L, Absolute basophil count: 0.28 × 10⁹/L. This profound eosinophilia prompted further investigation. NT-proBNP level was elevated at 4097.22 pg/mL, indicating severe cardiac strain. Stool examination showed no parasites or ova detected.

Peripheral blood smear findings indicated an increased neutrophil percentage, with toxic granules and vacuolar degeneration observed in the cytoplasm of some cells, along with marked eosinophilia. The neutrophil alkaline phosphatase stain showed a positivity rate of 30% and a score of 40. Bone marrow biopsy revealed hypercellularity with an elevated eosinophil proportion: granulocytic series (G) accounted for 79.60%, erythroid series (E) 13.20%, and a G/E ratio of 6.0:1. The granulocytic lineage was hyperplastic, demonstrating high eosinophil ratios across all maturation stages, while other stages exhibited normal ratios and morphology. The erythroid series was also hyperplastic, predominantly composed of intermediate and late erythroblasts with normal morphology, and mature erythrocytes appeared grossly normal.

Tumor marker levels were as follows: carcinoembryonic antigen: 6.26 μg/L; carbohydrate antigen CA125: 44.69 U/mL; neuron-specific enolase: 70.72 ng/mL; Chromosomal karyotype analysis revealed no abnormalities, and FGFR1 gene rearrangement was not detected. PDGFRA and PDGFRB gene testing could not be completed due to technical limitations with laboratory equipment.

Liver function, renal function, and electrolyte profiles showed no significant abnormalities. Serological testing for anti-extractable nuclear antigen antibodies, anti-neutrophil cytoplasmic antibodies (ANCA) including proteinase 3 (PR3), myeloperoxidase, and glomerular basement membrane antibodies revealed weakly positive anti-SSA/Ro antibodies and positive perinuclear ANCA (P-ANCA). The erythrocyte sedimentation rate was elevated at 100 mm/h, while rheumatoid factor and antinuclear antibodies were within normal limits.

**Figure 1.**
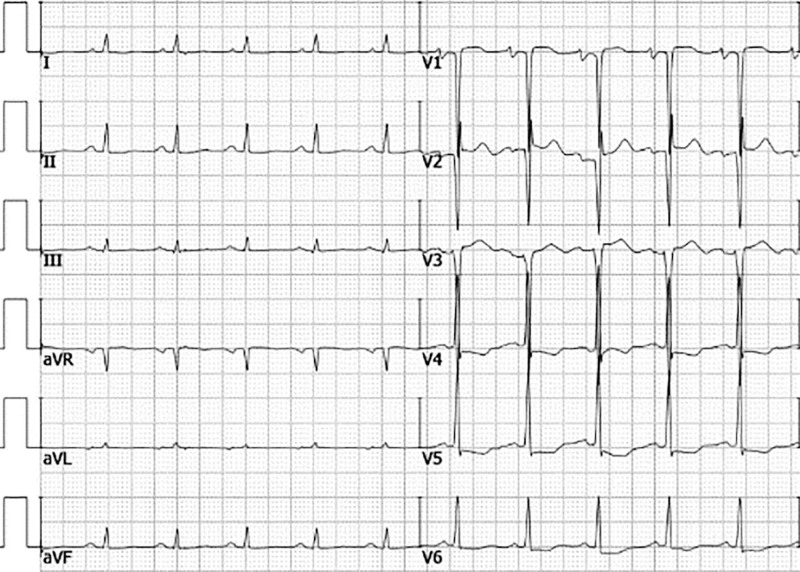
Admission electrocardiogram showing anterior ST-T segment changes.

**Figure 2.**
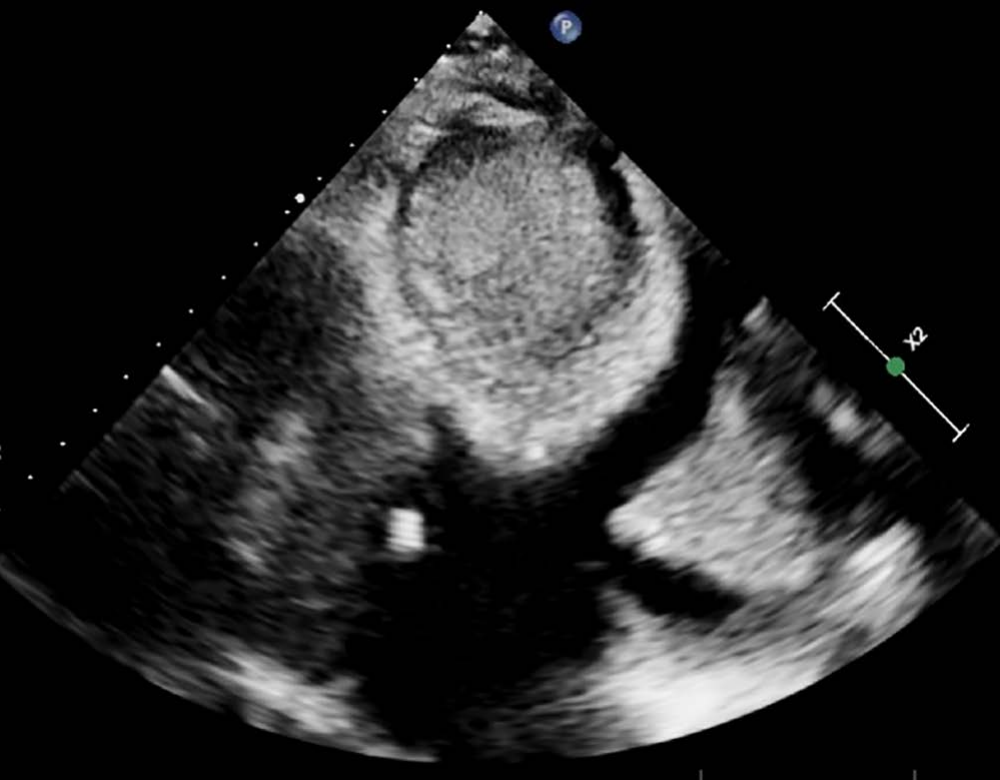
Transthoracic echocardiography demonstrating left ventricular apical obliteration.

**Figure 3.**
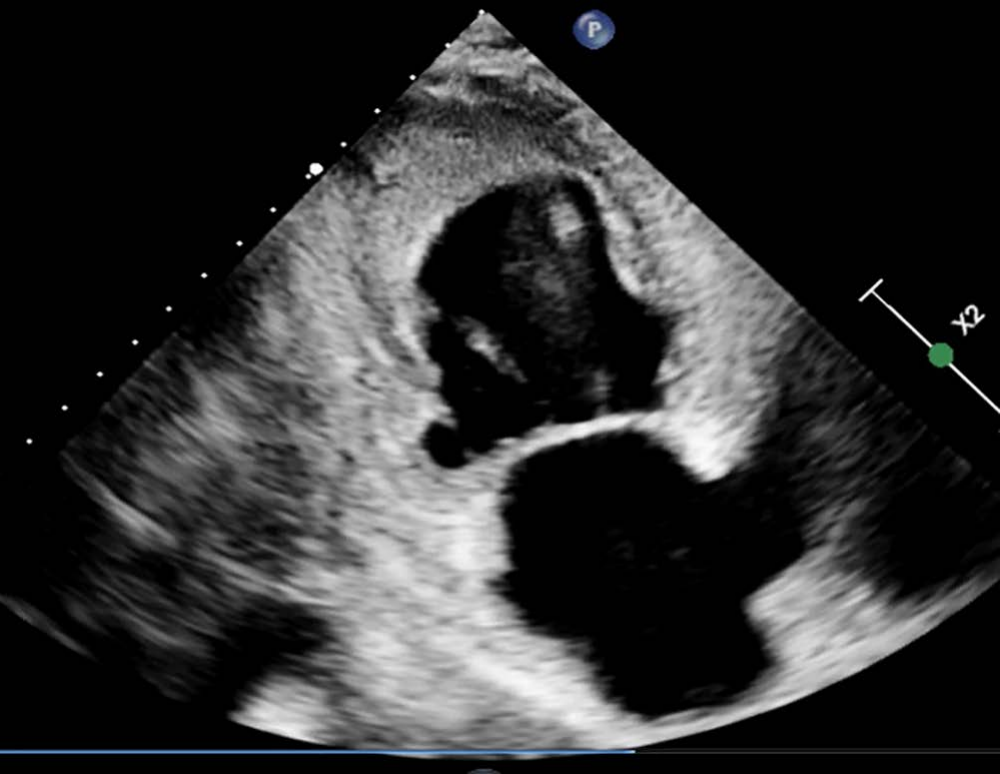
Echocardiogram showing left ventricular wall thickening with left atrial and ventricular enlargement.

The patient’s electrocardiogram revealed anterior ST-T segment changes (Fig. 1). Echocardiography demonstrated left ventricular endocardial thickening suggestive of Löffler endocarditis, thickened endocardium with intracardiac thrombus, and left atrial/ventricular enlargement (Figs. 2 and 3).

**Figure 4.**
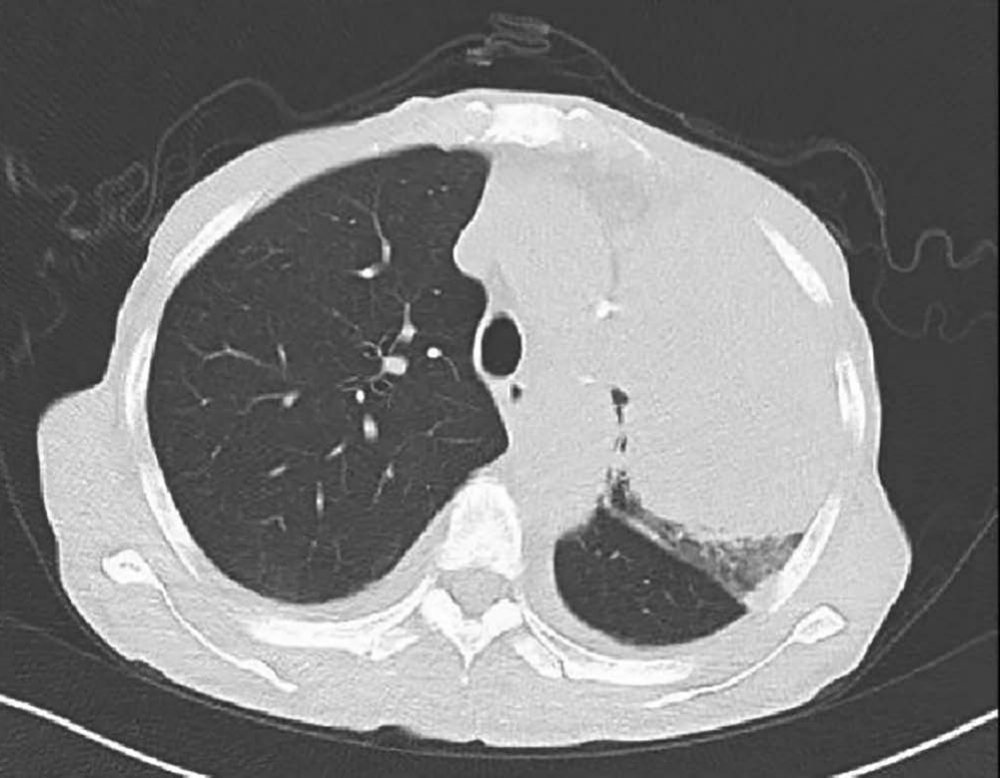
Initial chest computed tomography (CT) revealing a left upper lobe mass with bronchial obstruction.

**Figure 5.**
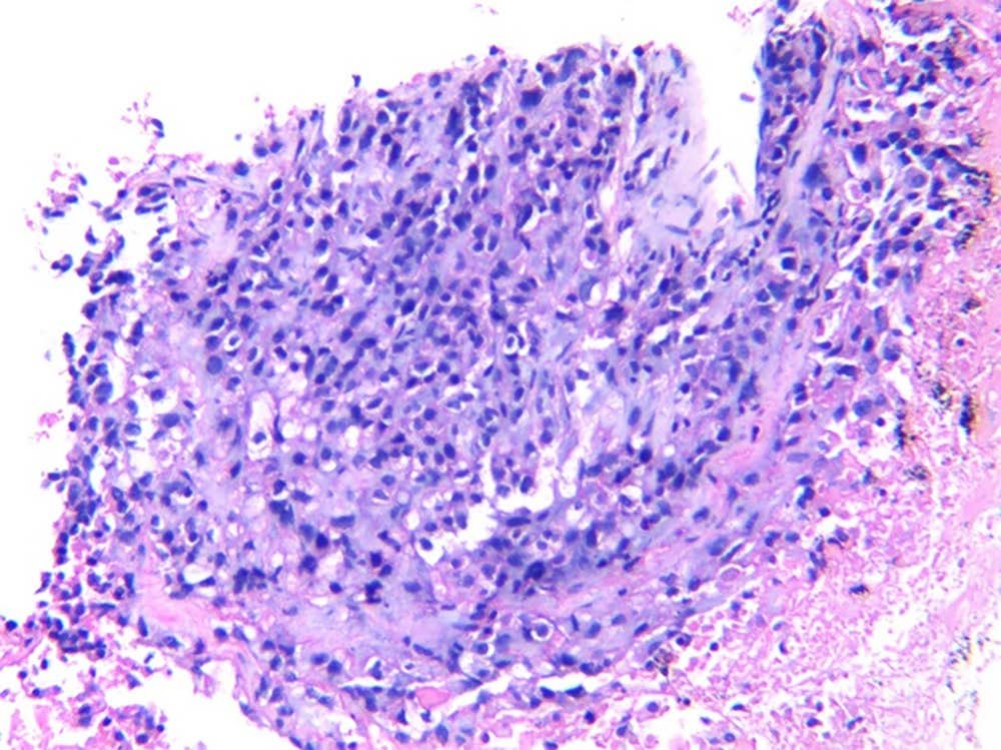
Lung biopsy specimen (HE × 400) showing nests of atypical epithelial cells consistent with adenocarcinoma.

Chest CT identified a mass-like high-density shadow in the left upper lobe with bronchial cutoff, raising suspicion for malignancy (Fig. 4). A lung mass biopsy revealed atypical cells arranged in nested clusters, consistent with non-small cell lung carcinoma. Immunohistochemical staining showed positivity for AE1/AE3, Vimentin, P63, P40, Napsin-A, and TTF-1, while Synaptophysin (Syn) and CD56 were negative. Ki67 proliferation index was approximately 30%, supporting a diagnosis of lung adenocarcinoma (Fig. 5).

The patient’s family declined corticosteroid therapy. Treatment included recombinant human brain natriuretic peptide (rhBNP) for vasodilation and cardiac load reduction, diuretics (furosemide and bumetanide), aminophylline for bronchodilation, and rivaroxaban for anticoagulation. Despite therapy, the patient’s clinical status remained unchanged. Following the lung biopsy confirming lung adenocarcinoma, the family opted to discontinue further treatment and requested voluntary discharge.

**Figure 6.**
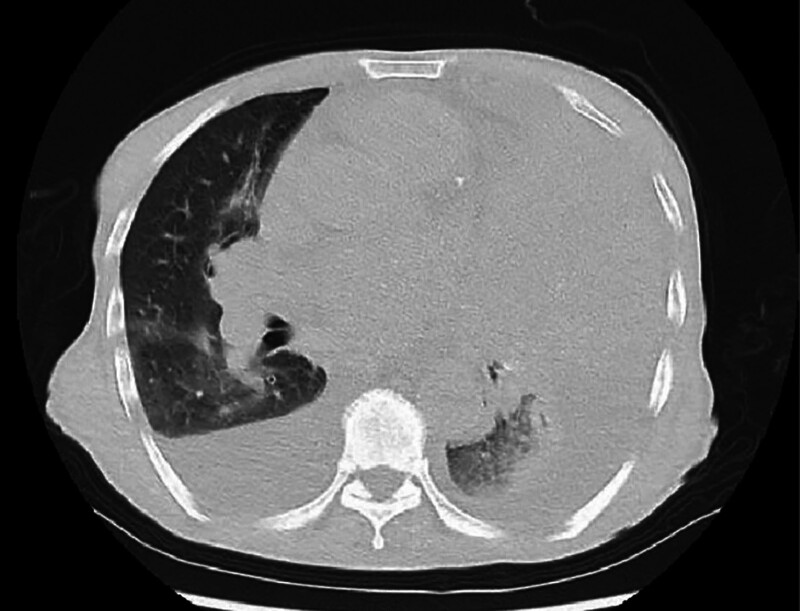
Follow-up chest CT demonstrating progression of left upper lobe mass with bilateral pleural effusions. CT = computed tomography.

In March 2023, the patient was readmitted with worsening dyspnea and bilateral lower limb edema. Repeat chest CT revealed a left upper lung mass, bilateral pleural effusions (significantly increased from prior imaging), and bilateral pneumonia (Fig. 6). Sputum culture identified Acinetobacter baumannii infection. Despite aggressive interventions, including broad-spectrum antibiotics, vasopressor support, nutritional supplementation, sputum suction, and endotracheal intubation with mechanical ventilation, the patient’s oxygen saturation remained unstable, and progressive hypotension ensued. The family ultimately withdrew care, and the patient was discharged voluntarily.

## 3. Discussion

HES is a rare disorder, with an estimated incidence of 0.4 per 1000,000 individuals according to SEER database.^[3]^ Eosinophilia (absolute eosinophil count > 500 cells/μL) is more common, with a incidence of 4%,^[4]^ often secondary to allergies, infections, drug reactions or hematologic tumor. HES in solid tumors is exceedingly rare, with only sporadic case reports describing this paraneoplastic phenomenon. While HES is classically linked to hematologic malignancies (e.g., chronic eosinophilic leukemia), its association with solid tumors remains poorly characterized. Lung adenocarcinoma, in particular, has been sporadically reported to drive paraneoplastic eosinophilia,^[5–7]^ In a review of Hypereosinophilia in solid tumors, 8 lung adenocarcinoma cases are reported with hypereosinophilia, average eosinophil count was 47.8 G/l with maximum cout of 114.39 G/l.^[8]^

The mechanism of eosinophilia in solid tumor remains incompletely understood but likely involves tumor-derived cytokines that stimulate eosinophil production. Among these, interleukin-5 (IL-5) plays a central role in eosinophilopoiesis by promoting differentiation, survival, and activation of eosinophils.^[9,10]^ Elevated IL-5 levels have been documented in lung carcinoma and correlate with eosinophil counts, decreasing only after tumor resection.^[11]^ GM-CSF and IL-2 may further amplify this effect^[12,13]^, as demonstrated by their presence in pleural fluid of affected patients.^[12]^

The presence of HES in cancer patients often heralds a poor prognosis. In lung adenocarcinoma, hypereosinophilia is associated with advanced disease, rapid progression, and resistance to conventional therapies,^[14]^ possibly due to systemic inflammation promoting tumor aggressiveness or diagnostic delays from overlapping symptoms (e.g., dyspnea attributed to cardiac vs pulmonary causes). In our case, the patient’s rapid decline underscores the challenges of managing dual pathologies with limited therapeutic overlap.

Therapeutic strategies for HES and Löffler endocarditis are etiology-driven, underscoring the importance of identifying the underlying cause. However, HES complicates oncology care by limiting treatment options and increasing toxicity risks. For HES secondary to hematologic malignancies, particularly those with PDGFRA or PDGFRB gene abnormalities, such as imatinib mesylate, a tyrosine kinase inhibitor is highly effective.^[15,16]^ Pemigatinib may benefit patients with FGFR1 alterations, though currently approved only for cholangiocarcinoma.^[17]^ Glucocorticoids remain first-line therapy for HES, though long-term use carries significant adverse effects.^[18]^ Secondary HES requires targeted therapy (e.g., antiparasitics).

For patients with concurrent heart failure, guideline-directed medical therapy should be initiated.^[19]^ Novel agents like vericiguat lack evidence in HES-related cardiac dysfunction. Anticoagulation is recommended for thromboembolic risk (e.g., intracardiac thrombi), with duration guided by thrombus resolution [6], its role in low-risk patients remains uncertain. Surgery or transplantation may be considered in refractory cases. Notably, corticosteroids, first-line therapy for HES, may impair immune responses to chemotherapy or immunotherapy, while targeted agents like imatinib are rarely applicable in solid tumors. Eosinophilia-induced organ damage (e.g., cardiac thrombosis) may also limit aggressive cancer therapy. In this patient, family refusal of corticosteroids and the advanced stage of malignancy precluded targeted HES management, hastening clinical deterioration.

This case highlights 3 key lessons: first, unexplained hypereosinophilia, particularly with cardiac involvement such as endomyocardial thickening or intracardiac thrombi, should prompt immediate evaluation for occult malignancy, even without respiratory symptoms. Second, solid tumors, including lung adenocarcinoma, must be considered in the differential diagnosis of hypereosinophilic syndrome, not just hematologic disorders. Third, early multidisciplinary collaboration between cardiology, oncology, and hematology is essential for timely diagnosis and optimal management. The educational value lies in its novelty: to our knowledge, this is the first report of Löffler endocarditis as a paraneoplastic manifestation of lung adenocarcinoma. It challenges the traditional view that cancer-associated eosinophilia is limited to hematologic malignancies and underscores the role of tumor-derived cytokines like IL-5 in driving systemic inflammation and end-organ damage. Recognizing this association may help avoid diagnostic delays and improve outcomes in a high-mortality condition.

This case underscores the need for heightened vigilance in patients with unexplained eosinophilia. Screening for occult malignancies, particularly lung cancer in smokers, is critical. Multidisciplinary collaboration between cardiologists, oncologists, and hematologists is essential to optimize care. Future research should explore cytokine-blocking therapies (e.g., anti-IL-5 monoclonal antibodies) to disrupt the paracrine pathways driving both HES and tumor growth.

## 4. Conclusion

Löffler endocarditis, as a rare entity, poses significant diagnostic challenges in clinical practice. Its high mortality and morbidity necessitate heightened clinician awareness to enable early detection, accurate diagnosis, and timely intervention. Improved recognition of this condition is critical to enhancing survival rates and quality of life for affected patients.

## Author contributions

**Conceptualization:** Hongmei Yao.

**Data curation:** Hongmei Yao, Yubin Chen.

**Investigation:** Yubin Chen.

**Methodology:** Yubin Chen.

**Resources:** Feng Bai.

**Software:** Chao Wu.

**Supervision:** Feng Bai.

**Validation:** Chao Wu.

**Visualization:** Zijing Wang.

**Writing – original draft:** Hongmei Yao.

**Writing – review & editing:** Zijing Wang, Feng Bai.
